# Biomechanical effects of altered multifidus muscle morphology on cervical spine tissues

**DOI:** 10.3389/fbioe.2025.1524844

**Published:** 2025-02-20

**Authors:** Guangming Xu, Chenxing Li, Zhizhong Sheng

**Affiliations:** ^1^ Department of Orthopaedics, Shenzhen PingleOrthopedic Hospital and Shenzhen Pingshan Traditional Chinese Medicine Hospital, Shenzhen, Guangdong, China; ^2^ Guangzhou University of Chinese Medicine, Guangzhou, Guangdong, China; ^3^ Pain Management, Shenzhen Guangming District People’s Hospital, Shenzhen, Guangdong, China

**Keywords:** multifidus muscle, cervical, intervertebral disc, finite element, joint capsules

## Abstract

**Background:**

Muscle fat infiltration and atrophy were common pathomorphologic changes in the paravertebral muscles. Some studies indicated that degeneration of paravertebral muscles may be one of the important causes of chronic neck pain. Therefore, we investigated the mechanical effects of multifidus muscle morphologic changes on cervical spine tissues by constructing cervical spine models of multfiidus muscle with different degrees of atrophy.

**Method:**

Three-dimensional finite element models of the cervical spine with 100%, 80%, and 50% with the multifidus muscle were constructed by referring to previous literature. According to the mechanical loading conditions in previous literature, the patient’s head weight and 1 Nm of loading were considered to be applied to the cervical spine, and the mechanical differences in the cervical intervertebral discs, joint capsule, cartilage endplates and range of motion (ROM) due to the morphological changes of the multifidus muscle were recorded and analyzed.

**Result:**

Under anterior flexion loading, model C increasing by 55% and 22% at the C5-6 segment compared to A and B, respectively. Among the three model groups, the stresses in the discs of the lower segments (C4-C7) were significantly higher than those in the upper segments. Under posterior extension loading, the strain values of the joint capsule were higher in the lower cervical segments, with the maximum strain values in the C5-6 segments. The maximum strain values in the lower cartilage endplates were in the C5-6 segments in model group A, whereas the maximum values were in the C4-5 segments in both models B and C. The maximum values in the lower cervical segments were in the C4-6 and C4-5 segments. In addition, a similar trend described above occurs in lateral bending and axial rotation conditions. The ROM of the lower cervical was higher than that of the upper cervical vertebrae, except in lateral bending conditions.

**Conclusion:**

In this study, we constructed the morphology of the multifidus muscle to more realistically simulate the mechanical environment of the cervical spine *in vivo* and quantitatively explored the effects of multifidus muscle atrophy on cervical spine tissues. The results showed that volume atrophy of the multifidus muscle altered the mechanical response of cervical spine tissues. Volume atrophy of the multifidus muscle significantly increased the mechanical indexes of the cervical spine tissues, in which the cervical disc stresses, joint capsule strains, and cartilage endplates increased significantly. Compared with the mechanical changes in the upper cervical segments, the mechanical changes in the lower cervical segments were higher. Therefore, it is important to moderately increase the functional exercise of the multifidus muscle to prevent atrophy leading to abnormal stress concentrations in cervical tissues.

## 1 Introduction

Muscle fat infiltration and atrophy were common pathomorphological changes in the paravertebral muscles and are associated with various spinal disorders (Huang et al., 2022; Shi et al., 2022; Suo et al., 2023). Degeneration of the paravertebral muscles may be an important factor in chronic neck pain and cervical rehabilitation (Li et al., 2024; Naghdi et al., 2023). Patients with cervical spondylosis had a greater degree of fatty infiltration and sagittal imbalance in the multifidus muscle compared to healthy subjects, which correlated with cervical spine pain and injury (Li et al., 2023). In addition, intervertebral disc degeneration was associated with fatty infiltration of the paraspinal muscles, especially of muscles such as the multifidus muscle (Li et al., 2024). The deep extensors, composed of muscles such as the multifidus, play an important role in maintaining sagittal balance in the cervical spine and correlate with the severity of neck symptoms (Gu et al., 2023). The cervical multifidus muscle CSA was generally smaller in women with bilateral chronic neck pain than healthy women (Fernández-de-las-Peñas et al., 2008). Thus, morphological changes in the multifidus muscle may be important in cervical spondylosis.

The results showed a correlation with reduced sensorimotor function in cervical spondylosis by calculating the amount of fat infiltration in the bilateral multifidus muscles on MRI images (Cloney et al., 2018). Alterations in muscle composition may reduce the ability to produce or maintain muscle strength, and may contribute to chronic pain (Snodgrass et al., 2024). Strengthening the paraspinal muscles is one of the most effective ways to reduce neck pain due to fatty infiltration and volume changes in the paraspinal muscles. Kang et al. (2021) suggested that increasing the volume of the paraspinal muscles through strengthening exercises can reduce the load on the spine and have a pain-relieving effect. Due to the complexity of the *in vivo* structure of the spine, studies on morphological changes in the multifidus muscle that affect neck tissues were limited.

In this study, we simulated the morphological changes of the multifidus muscle by constructing cervical spine models with different degrees of atrophy, and quantified the mechanical effects on cervical spine tissues. The mechanical differences in cervical intervertebral discs, joint capsules, cartilage endplates and mobility were analyzed by loading the cervical spine in different working conditions with multifidus muscle morphological changes.

## 2 Methods

### 2.1 Model construction

**FIGURE 1:**
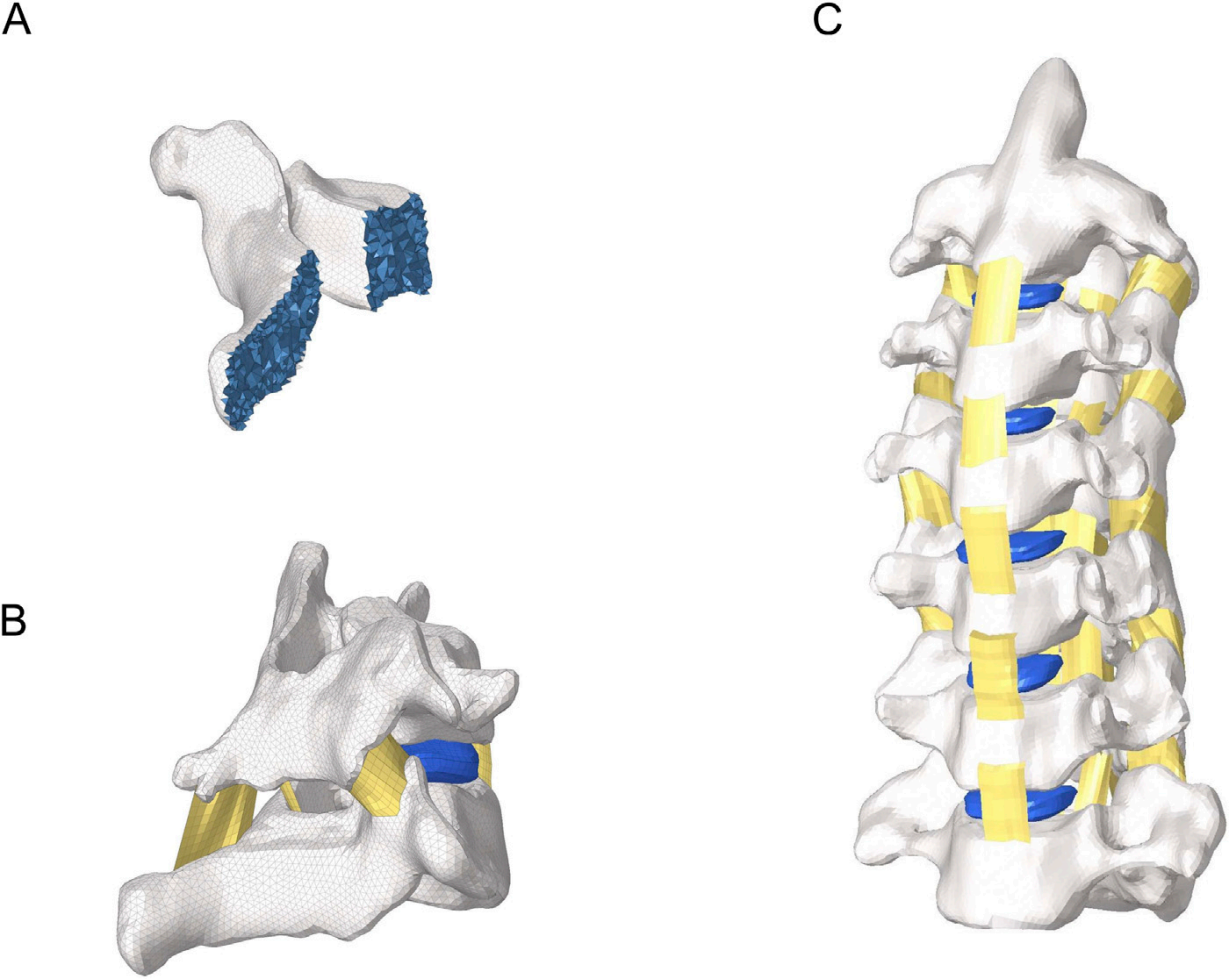
Overall view of the cervical spine model. **(A)** Internal view of the vertebral body. **(B)** Intersegmental ligaments. **(C)** Lateral view of cervical spine model.

A finite element model of the cervical spine was constructed based on a healthy male volunteer, which referred to the modeling approach of previous study (Chen et al., 2024), and the intervertebral discs were constructed in the model was improved, as shown in Figure 1. A cross-sectional scan of the cervical spine of the volunteer was performed using spiral CT and the images were saved in DICOM file format. The DICOM file was imported into Mimics for 3D reconstruction, and the C2-C7 cervical vertebral segment model was extracted using tools such as masking and filling, after which the model was saved and imported into Geomagic, and the model was initially adjusted and polished using tools such as deletion of pegs, relaxation, noise reduction, and other tools, and then processed by delineating the whorl lines and constructing the surface pieces. The model was imported into SolidWorks to construct the intervertebral disc structure. Hypermesh software was used to construct and mesh the components such as the joint capsule, endplates, and annulus fibrosus, with a mesh size of 1 mm, and to assign each structural parameter to the model as shown in Table 1 (Sun et al., 2022; Wang et al., 2016; Purushothaman et al., 2021; Schmidt et al., 2007). Cortical bone and Bony endplate were simulated using Triangular shell with Isotropic elastic-plastic material. Intervertebral discs were simulated constructed using their Hexahedral with Mooney-Rivlin material properties. Annulus fibrosus fibers were defined as Orthotropic nonlinear elastic and ligaments were defined as Non-linear curves.

**TABLE 1:** Material properties of cervical tissue.

Component	Element type	Material type	Material parameters
Cortical bone	Triangular shell	Isotropic elastic–plastic	E = 10.0 GPa v = 0.3
Cancellous bones	Tetrahedral		E = 300 MPa v = 0.3
Bony endplate	Triangular shell		E = 5.6 GPa v = 0.3
Nucleus pulpous	Hexahedral	Mooney-Rivlin	c10 = 0.12, c01 = 0.03
Annulus fibrosus matrix			c10 = 0.18, c01 = 0.045
Annulus fibrosus fibers	Quadrilateral membrane	Orthotropic nonlinear elastic	N/A
Ligaments		Non-linear curves	N/A

N/A, Not applicable.

### 2.2 Constructing finite element models of different degrees of atrophy of the multifidus muscle

**FIGURE 2:**
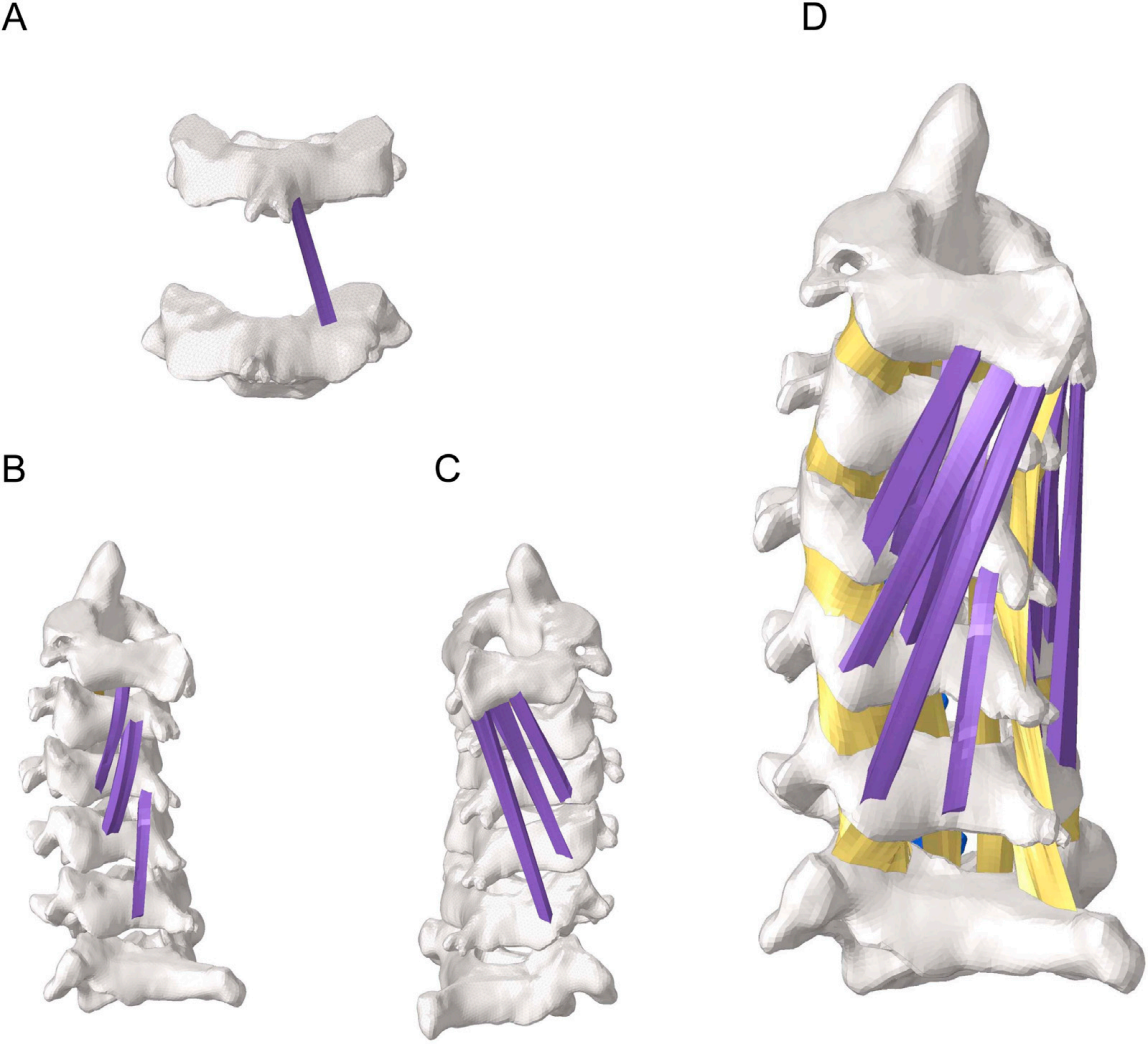
Finite element model of the cervical spine with the multifidus muscle. **(A)** View of part of the deep multifidus muscle. **(B)** Lateral view of the deep multifidus muscle. **(C)** Lateral view of the superficial multifidus muscle. **(D)** A holistic view of the cervical spine including the multifidus muscle.

**FIGURE 3:**
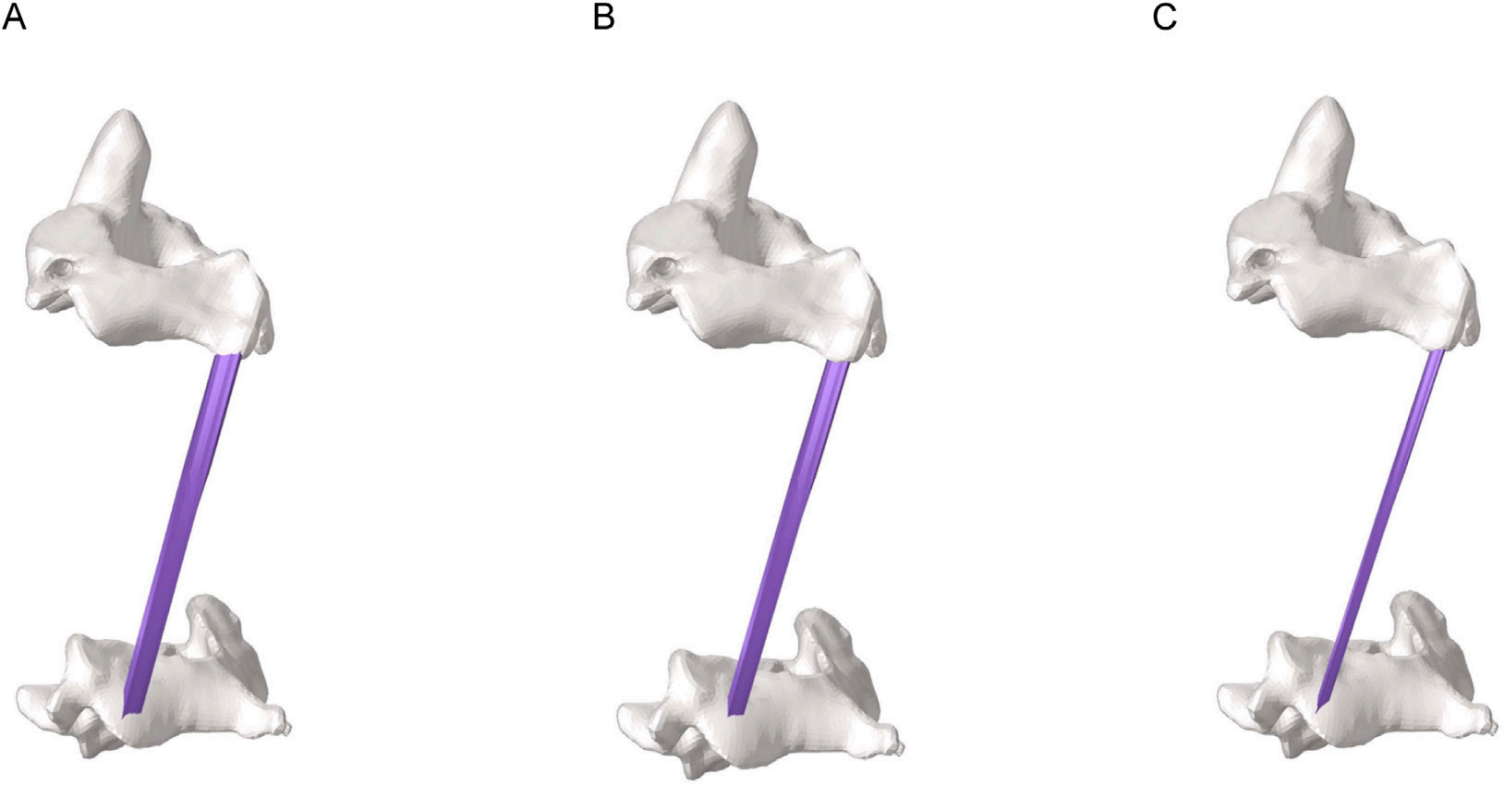
Partial view of multifidus muscle atrophy. **(A)** Finite element model with 100% content of multifidus muscle. **(B)** Finite element model with 80% content of multifidus muscle. **(C)** Finite element model with 50% content of multifidus muscle.

To assess the effects of morphological changes of the multifidus muscle on the cervical spine tissues, a finite element model of the cervical spine containing the multifidus muscle was constructed in Figure 2 (Anderson et al., 2005). Referring to previous literature (Kang et al., 2021), finite element models with 100%, 80% and 50% content of multifidus muscle were established in Figure 3. Among them, the multifidus muscle was defined as nonlinear hyperelastic and incompressible (Dao et al., 2014). The contact between the multifidus muscle and the bone was set to be bound.

### 2.3 Loading conditions

In all the cervical spine models, C7 and the lower end of the multifidus muscle were set to be completely fixed. According to the mechanical loading conditions in previous literature (Sun et al., 2023), the patient’s head weight and 1 Nm load were applied to the cervical spine. The segmental range of motion in the model was calculated and compared with the literature, which was used to verify the validity of the model. Then, based on the above mechanical loading conditions, the mechanical changes of the cervical spine tissues by atrophy of the multifidus muscle were recorded and analyzed in different working conditions.

## 3 Results

### 3.1 Validation of cervical model

**FIGURE 4:**
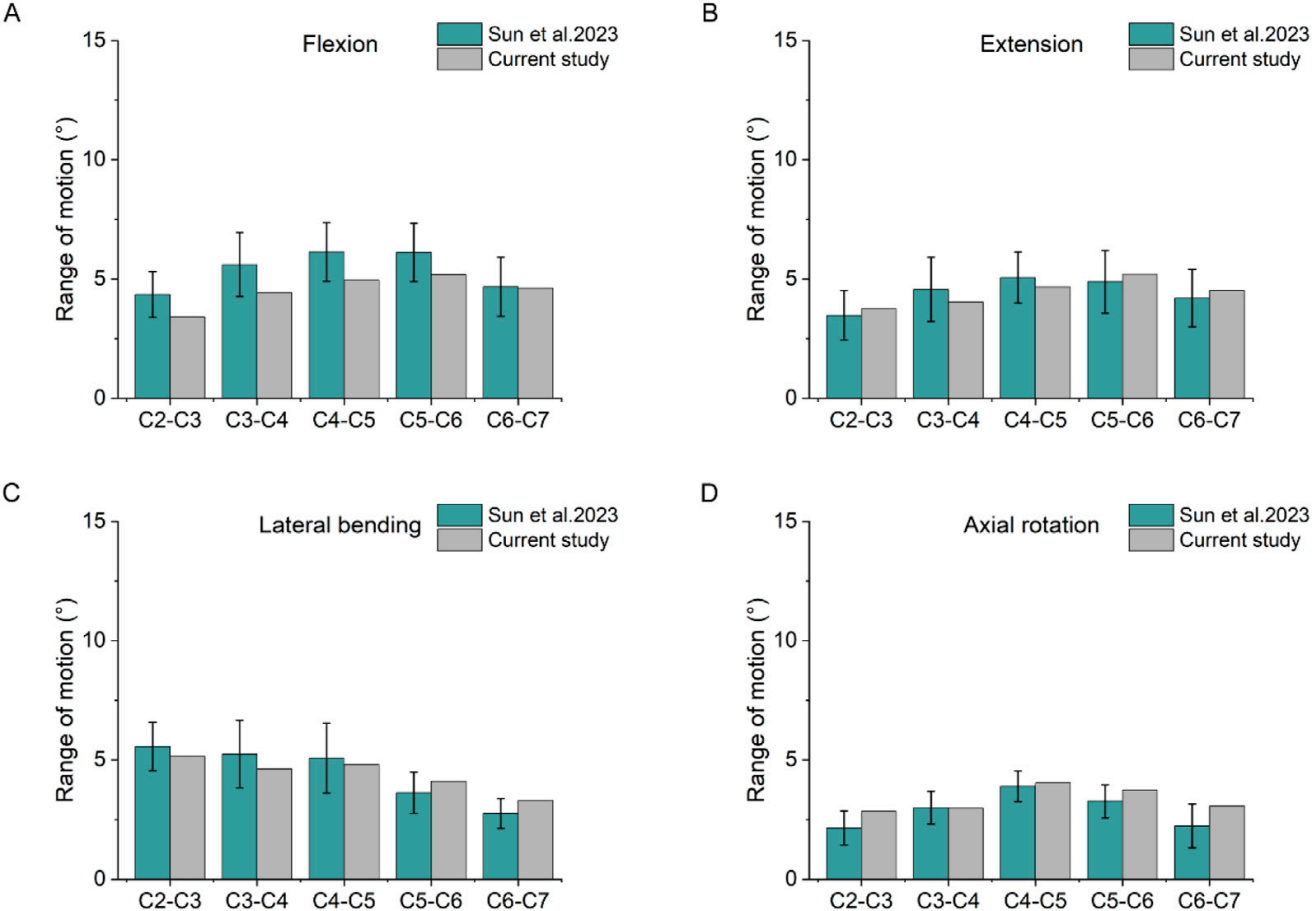
Comparison of ROM. **(A)** Results of the ROM under flexion. **(B)** Results of the ROM under extension. **(C)** Results of the ROM under lateral bending. **(D)** Results of the ROM under axial rotation.

The results of the cervical spine model were compared with the data of Sun et al. (2023) by calculating the ROM results of the cervical spine model in the four working conditions of forward flexion, backward extension, lateral flexion and axial rotation. The results were shown in Figure 4, and the trend and range of the model results were in good agreement with the data of Sun et al. (2023).

### 3.2 Changes in intervertebral disc stress

**FIGURE 5:**
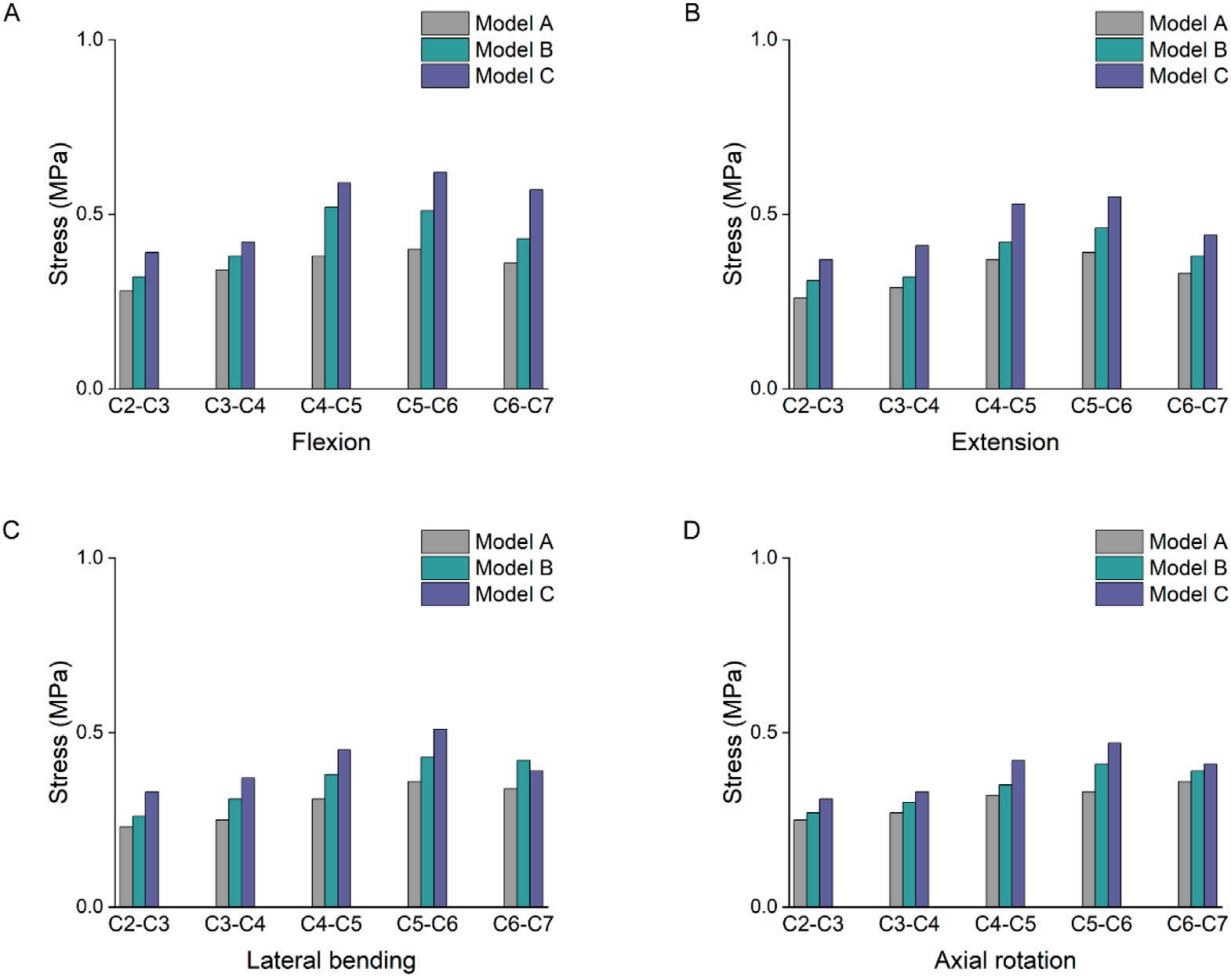
Intervertebral disc stress result. **(A)** Stress results of the intervertebral disc under flexion. **(B)** Stress results of the intervertebral disc under extension. **(C)** Stress results of the intervertebral disc under lateral bending. **(D)** Stress results of the intervertebral disc under axial rotation.

Figure 5 showed the stress values of cervical intervertebral disc stress under different working conditions. Under forward flexion loading, the stress changes of the three models were more obvious. Model C had the highest stress, which, at the C5-6 segment, increased by 55% and 22% compared with models A and B, respectively. Under posterior extension loading, the stress values of the C5-6 intervertebral discs of the three models increased by 41% and 20% in Model C compared with those of models A and B, respectively. Under lateral bending and axial rotation conditions, the intervertebral disc stress values at C4-5 and C5-6 segments were greater than those at other segments. In the three models, the stresses in the discs of the lower segments (C4-C7) were significantly higher than those in the upper segments.

### 3.3 Strain changes of joint capsules

**FIGURE 6:**
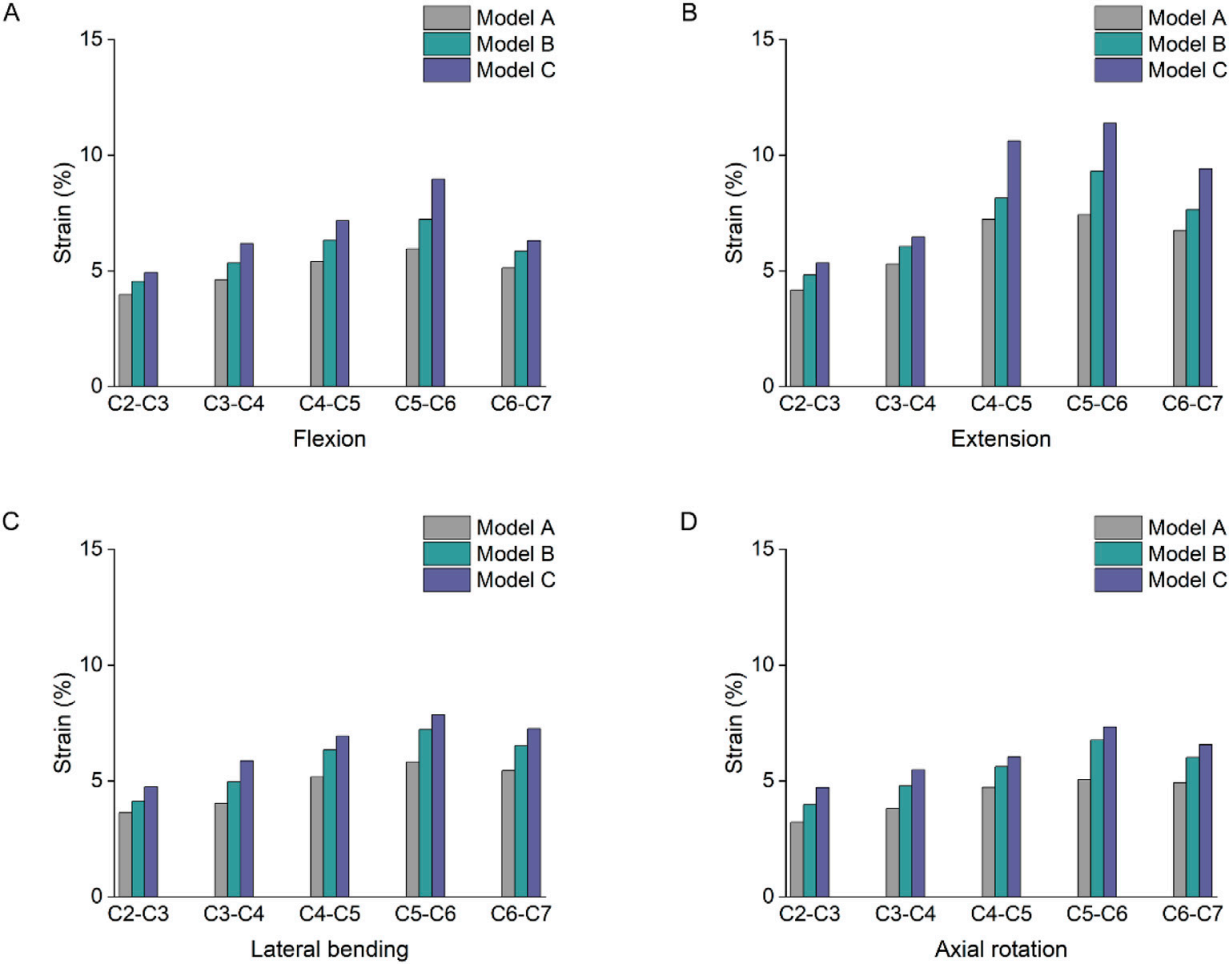
Strain changes of joint capsules. **(A)** Strain changes of joint capsules under flexion. **(B)** Strain changes of joint capsules under extension. **(C)** Strain changes of joint capsules under lateral bending. **(D)** Strain changes of joint capsules under axial rotation.

As shown in Figure 6, the joint capsule strain varied from C2 to C7, and the stress distribution was not uniform. Under flexion loading, the joint capsule strain value of model C was higher, compared with models A and B, C4-5 increased by 33% and 13% respectively, and C5-6 increased by 51% and 24% respectively. Under extension condition, the strain values of the joint capsule in model C were greater than those in models A and B. The strain values of the joint capsule in the lower cervical segments were significantly higher. The C5-6 segment with the greatest strain value, which increased by 53% and 22%, respectively, compared with models A and B. Under lateral bending and axial rotation conditions, the capsular strain values of C4-5, C5-6 and C6-7 segments were higher than those of C2-3 and C3-4 segments.

### 3.4 Stress results of the lower cartilage endplates

**FIGURE 7:**
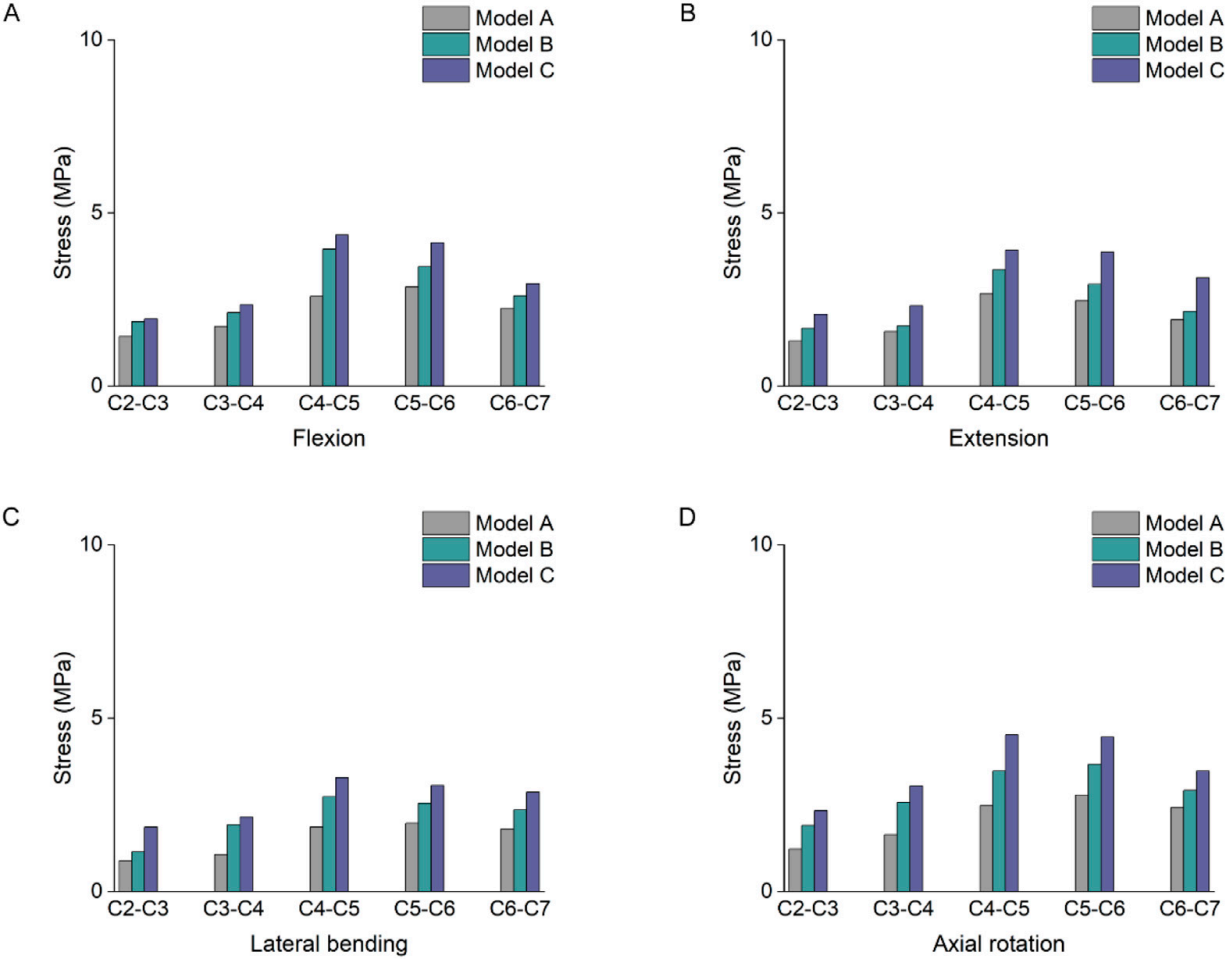
Stress results of the lower cartilage endplates. **(A)** Stress results of the lower cartilage endplates under flexion. **(B)** Stress results of the lower cartilage endplates under extension. **(C)** Stress results of the lower cartilage endplates under lateral bending. **(D)** Stress results of the lower cartilage endplates under axial rotation.

The stress results of the lower cartilage endplates in the three groups showed an increasing trend change in Figure 7. Among them, the maximum stress value in model A was 2.87 MPa at segment C5-6, while the maximum value in both models B and C was at segment C4-5. At the C4-5 segment under flexion loading, the stress in model C increased by 69% and 10% compared to models A and B, respectively. As the degree of multifidus atrophy increased, it altered the stress distribution in the cervical segments and led to stress concentrations in the lower cartilage endplates.

### 3.5 Comparison of ROM

**FIGURE 8:**
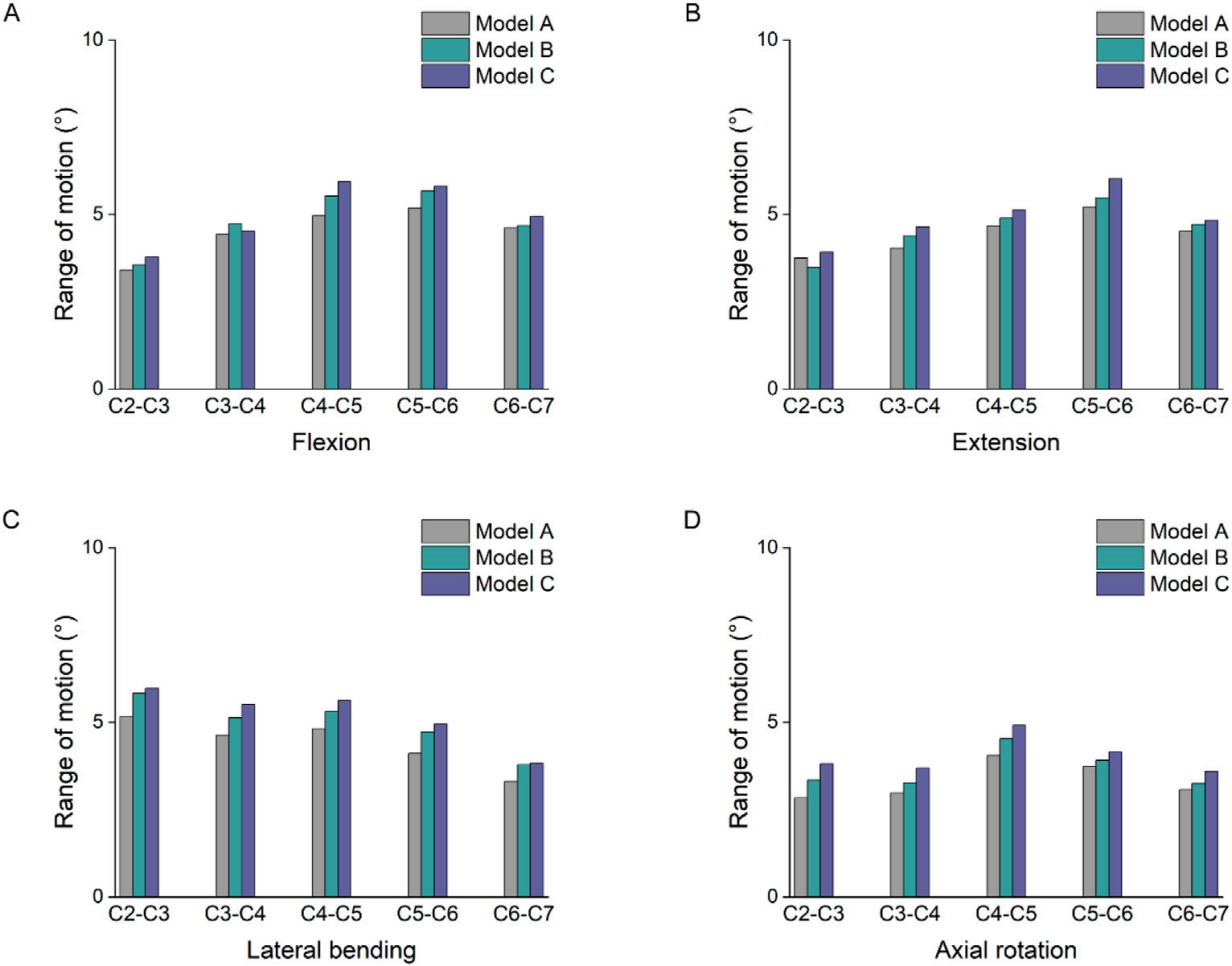
Comparison of ROM. **(A)** Results of the ROM under flexion. **(B)** Results of the ROM under extension. **(C)** Results of the ROM under lateral bending. **(D)** Results of the ROM under axial rotation.

Figure 8 showed significant differences in the ROM of the segments with increasing degrees of multifidus atrophy. Under flexion and extension loading, the increase in cervical spine ROM was significantly more pronounced in the lower segments than in the upper segments. The largest activity angles in the three models were at the C4-5 and C5-6 segments. In particular, under flexion loading, there was an 18% and 8% increase in C5-6 in model C compared with models A and B, respectively. Under extension loading, the C5-6 segments increased by 15% and 4% in model group C compared to models A and B, respectively. In addition, a similar trend described above occurs in lateral flexion and rotation conditions.

## 4 Discussion

In the present study, we found that increased multifidus muscle atrophy altered mechanical indices such as cervical disc stress, joint capsule strain, and cartilage endplate stress, and to varying degrees with increased multifidus muscle atrophy. In addition, changes in cervical ROM are most obvious in the lower segments with multifidus atrophy.

The paraspinal muscles are a series of skeletal muscles that maintain the motor function and stability of the cervical spine (Suo et al., 2024). Measurement of muscle CSA is an important indicator of fundamental changes in muscle structure, particularly concerning muscle strength and atrophy (De Pauw et al., 2016; Fortin et al., 2018; Airi et al., 2008). The mechanical effects of multifidus atrophy on cervical spine tissues were quantitatively analyzed through a finite element approach, and our results suggest that normal cervical multifidus muscles efficiently distribute loads applied to the cervical spine, and that, with atrophy, the loads may be excessively concentrated on certain intervertebral discs or joint capsule. These results are consistent with previous research that fatty infiltration of the cervical multifidus muscle can cause cervical injury and mechanical neck pain (Li et al., 2023). Deep neck muscle weakness and associated atrophy may be a risk factor for the emergence and recurrence of neck pain (Amiri-Arimi et al., 2020). This may be due to the fascicular attachment pattern of the multifidus muscle in the cervical region, which is directly attached to the small cervical joint capsule in a manner that suggests an interaction between the multifidus muscle and the joint capsule (Anderson et al., 2005). Therefore, clarification of the mechanical changes in cervical spine tissues due to atrophy of the multifidus muscle was important for subsequent prevention and treatment.

Segmental mechanical dysfunction due to defects in the structure and activation of the multifidus may result in cumulative damage to the annulus fibrosus, which has been shown to lead to disc degeneration (Maas et al., 2018). In addition, intervertebral disc degeneration may lead to fibrosis of the multifidus muscle and structural changes in the muscle spindle (James et al., 2024; James et al., 2022). The muscle spindle of the multifidus muscle is mainly concentrated near the vertebral plate, and when the multifidus muscle atrophies, there may be a decrease in the stability of the cervical spine, which in turn affects mobility (Boyd-Clark et al., 2002). In addition, atrophy or reduced function of the multifidus muscle may directly increase segmental mechanical overload and affect spinal stability (Ignasiak et al., 2018). Our results suggest that the increase of lower segment motion caused by multifidus muscle atrophy also affects spinal stability.

Physiological degeneration of the cervical multifidus muscle tissue may be considered a potential pathological marker of cervical spondylosis (Li et al., 2023). Altering the physical properties of neck musculature can change its function (Thakar et al., 2014), which is similar to our results that polydactyly atrophy increases stress and strain in cervical spine tissues. Increased stress and strain increase the risk of tissue degeneration and injury and increase the occurrence of mechanical neck pain, which is one of the common symptoms of cervical spondylosis. Recent Studies (Li et al., 2023; Li et al., 2025) have pointed to a significant relationship between paraspinal muscle degeneration and the progression of cervical spondylosis. As only the effects of multifidus muscle atrophy on cervical spine tissues were included in this study, in subsequent studies, more complete neck muscles should be constructed and combined with skeletal muscle dynamics to simulate the mechanical effects of multifidus muscle atrophy on cervical spine tissues under active muscle forces.

The following deficiencies still exist in this paper. Firstly, the construction of the finite element model of the cervical spine is not complete enough, lacking nerves, blood vessels, other muscle tissues, etc., and a more complete model should be constructed in the subsequent research; Then, the multifidus muscle tissue constructed in this paper is a passive structure and an active muscle force model should be incorporated into the subsequent research; Furthermore, the construction of multifidus muscle atrophy in this study is isometric atrophy, however, the muscle atrophy in the clinic will lead to changes in muscle stiffness and fiber type, etc. However, due to the technical limitations of the model construction, which led to a certain error between the calculation results and the real situation, future studies should establish a more realistic and accurate finite element model and further validate its analysis results in the clinic.

## 5 Conclusion

This study indicates that multifidus atrophy alters the mechanical response of cervical spine tissues. The more severe the degree of multifidus atrophy, the more pronounced the increase in cervical disc stress, joint capsule strain, and cartilage endplates, and resulted in increased cervical spine mobility in the lower segments. Changes in the above outcomes increase the risk of injury by increasing the stresses on the cervical spine tissues. Therefore, it is important to moderately increase functional exercises targeting the multifidus muscle in the clinic in order to prevent atrophy that can lead to abnormal concentrations of stress in cervical spine tissues.

## Data Availability

The original contributions presented in the study are included in the article/supplementary material, further inquiries can be directed to the corresponding author.
